# Umbelliferone Ameliorates Benign Prostatic Hyperplasia by Inhibiting Cell Proliferation and G1/S Phase Cell Cycle Progression through Regulation of STAT3/E2F1 Axis

**DOI:** 10.3390/ijms22169019

**Published:** 2021-08-21

**Authors:** Hyo-Jung Kim, Bo-Ram Jin, Hyo-Jin An

**Affiliations:** Department of Pharmacology, College of Korean Medicine, Sang-ji University, Wonju-si 26339, Korea; hyojung_95@naver.com (H.-J.K.); wlsqh92@gmail.com (B.-R.J.)

**Keywords:** androgen receptor, benign prostatic hyperplasia, E2F transcription factor 1, signal transducer and activator of transcription 3, testosterone propionate, umbelliferone

## Abstract

Umbelliferone (UMB), also known as 7-hydroxycoumarin, is a derivative of coumarin, which is widely found in many plants such as carrots, coriander, and garden angelica. Although many studies have already revealed the various pharmacological properties of UMB, its effect on benign prostatic hyperplasia (BPH) remains unclear. Therefore, the present study aimed to elucidate the underlying mechanism of the anti-proliferative effect of UMB in a human benign prostatic hyperplasia cell line (BPH-1), as well as its ameliorative effect on BPH in testosterone propionate (TP)-induced rats. The results showed that UMB exerts an anti-proliferative effect in BPH-1 cells by modulating the signal transducer and activator of transcription 3 (STAT3)/E2F transcription factor 1 (E2F1) axis. UMB treatment not only inhibited androgen/androgen receptor (AR) signaling-related markers, but also downregulated the overexpression of G1/S phase cell cycle-related markers. In TP-induced rats, UMB administration demonstrated an anti-BPH effect by significantly reducing prostate size, weight, and epithelial thickness. In addition, UMB suppressed cell proliferation by reducing the expression of proliferating cell nuclear antigen (PCNA) and p-STAT3 (Tyr 705) in prostate tissue following TP injection. These findings suggest that UMB has pharmacological effects against BPH.

## 1. Introduction

Benign prostatic hyperplasia (BPH), a common disorder that occurs in older men, is characterized by uncontrolled proliferation of the prostate, an encapsulated accessory reproductive gland surrounding the bladder neck and proximal urethra [1]. An enlarged prostate gradually compresses the urethra, resulting in lower urinary tract symptoms (LUTS), such as a weak stream, residual urine, urinary frequency, urgency, and nocturia; consequently, it negatively impacts the quality of life of the patient [2]. Although various risk factors, including hormonal changes, aging, and growth factors, have been hypothesized to be associated with the onset and progression of BPH, a consensus on the etiology of BPH has not yet been reached [3].

Dihydrotestosterone (DHT), a form of circulating testosterone converted by 5α-reductase (5AR) presents in the prostate, plays an important role in the development of BPH [4]. Finasteride (Fina), a 5AR-inhibitor (5ARI) that is used to treat symptomatic BPH by preventing the conversion of testosterone to DHT, relieves dysuria by reducing the size of the enlarged prostate [5]. Unfortunately, numerous clinical studies have reported that 5ARI therapy causes adverse effects such as decreased libido, impaired ejaculation, and erectile dysfunction [6]. For this reason, the need for natural medicines to replace existing synthetic drugs is emerging [7]. Saw palmetto (Saw), an extract from the fruit of the American dwarf palm tree, *Serenoa repens*, is a relatively stable botanical agent used against the side effects of chemotherapy [8]. It is widely used in the treatment of LUTS and acts by inhibiting 5AR via a mechanism similar to that of Fina, but does not effectively reduce prostate size [9]. Therefore, there is a pressing need to identify alternative agents with reduced side effects that are capable of exerting the anti-BPH effect and determine their molecular mechanisms. Androgen receptor (AR), a member of the nuclear receptor superfamily, is a ligand-responsive transcription factor that mediates signaling by androgens, including DHT [10]. The transcriptional activation of AR triggers the transcription of target genes responsible for cell proliferation, along with co-regulators such as the steroid receptor coactivator (SRC) family, SRC-1 [11]. Therefore, targeting the androgen/AR signaling pathway is a major therapeutic approach against BPH.

Unregulated cell proliferation and reduction of apoptosis cause an increase in the total number of prostate cells during progression of BPH [12]. The division and replication of prostate cells are governed by the cell cycle consisting of four phases, among which failure of the G1/S checkpoint causes uncontrolled DNA replication, leading to aberrant cell proliferation [13]. The transition from G1 to S phase is primarily regulated by the cyclin–cyclin-dependent kinase (Cdk) complex that phosphorylates retinoblastoma (Rb), with the liberation of E2F transcription factors [14]. Signal transducer and transcription activator 3 (STAT3) is a member of the STAT family and is expressed in most tissue types. It is a latent cytoplasmic transcription factor that has a dual molecular role as a signal transducer and transcriptional activator [15]. Upon tyrosine phosphorylation by stimuli such as cytokines or growth factors, STAT3 is translocated into the nucleus where it regulates the transcription of target genes, thereby participating in several cellular processes, including cell proliferation and differentiation [16].

Umbelliferone (UMB) is a synthetic form of coumarin, a natural phenolic constituent widely distributed in the Rutaceae and Apiaceae (Umbelliferae) families [17]. UMB, which has excellent nutritional value, is commonly found in numerous fruits, vegetables, and plants (e.g., carrots, coriander, garden angelica) [18]. In previous studies, UMB has been reported to have antioxidant, anti-inflammatory, and anti-tumor effects [19,20,21]. Owing to its wide range of pharmacological and biological activities, UMB has drawn considerable attention as an attractive phytochemical in the fields of nutritional supplements, functional foods, and pharmaceuticals. Recently, the anti-carcinogenic effect of UMB was demonstrated in the prostate, but its effect on BPH has not yet been elucidated [22]. Hence, this study aimed to evaluate the potential of UMB as a therapeutic agent for BPH by identifying the effect of UMB on dysregulated cell proliferation and G1/S phase cell cycle progression in BPH-1 cells and TP-induced rats.

## 2. Results

### 2.1. Effect of UMB on the Blockade of Androgen/AR Signaling in BPH-1 Cells

**Figure 1:**
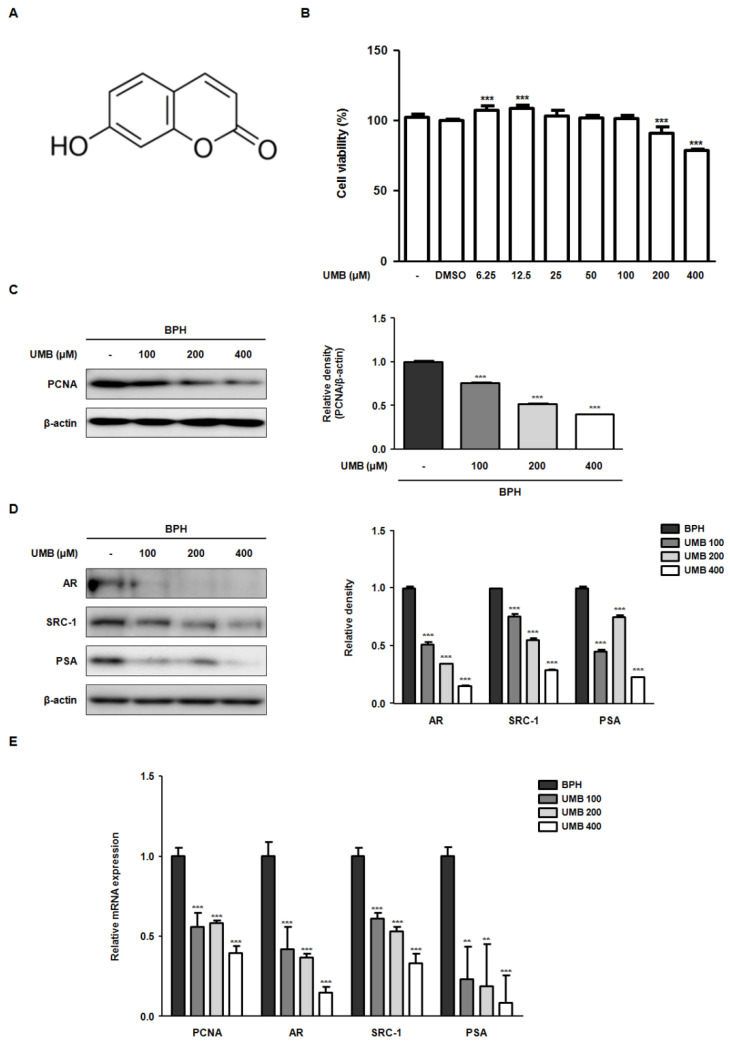
Effect of UMB on the blockade of androgen/AR signaling in BPH-1 cells. (**A**) Molecular structure of UMB. (**B**) BPH-1 cells were treated with or without different concentrations (6.25–400 μM) of UMB for 24 h, and cell viability was assessed. (**C**,**D**) BPH-1 cells were treated with or without 100, 200, and 400 μM of UMB. The protein expression of (**C**) PCNA, (**D**) AR, SRC-1, and PSA was determined by Western blot analysis using specific antibodies. The relative density of the proteins was normalized to that of β-actin, which was used as an internal control. (**E**) The mRNA expression of PCNA, AR, SRC-1, and PSA was quantified using qRT-PCR in BPH-1 cells. The relative mRNA expression was normalized using the Ct value of β-actin. The values represent as mean ± standard deviation (S.D.) of three independent experiments. ** *p* < 0.01, *** *p* < 0.001 vs. vehicle control.

To evaluate the inhibitory effect of UMB on cell proliferation, an MTT assay was performed using BPH-1 cells derived from the prostate epithelium of an older man with BPH. BPH-1 cells were treated with various concentrations (6.25–400 μM) of UMB for 24 h. The growth of BPH-1 cells was significantly hampered by UMB concentrations above 200 μM (Figure 1B). In addition, to validate the anti-proliferative effect of UMB, the expression of proliferating cell nuclear antigen (PCNA) was confirmed through Western blot analysis. Treatment with UMB (100, 200, and 400 μM) reduced PCNA protein expression in a concentration-dependent manner (Figure 1C). Activated AR signaling has a causative role in BPH progression by inducing the production of androgen-regulated genes, such as PSA, and promoting prostate growth [23]. To examine whether UMB interferes with androgen/AR signaling, the expression of androgen-related genes was detected. Protein expression of AR, SRC-1, and PSA, which was elevated in BPH-1 cells, was reduced following UMB treatment (Figure 1D). Similar to the results of Western blot analysis, UMB treatment of BPH-1 cells markedly downregulated the mRNA expression of PCNA, AR, SRC-1, and PSA (Figure 1E).

### 2.2. Effect of UMB on G1/S Phase Cell Cycle Arrest in BPH-1 Cells

**Figure 2:**
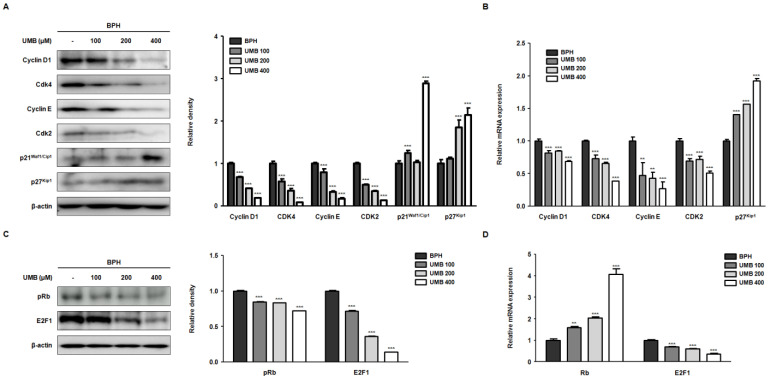
Effect of UMB on cell cycle arrest at the G1/S phase in BPH-1 cells. (**A**–**D**) BPH-1 cells were treated with or without 100, 200, and 400 μM of UMB. The protein expression of (**A**) cyclin D1, Cdk4, cyclin E, Cdk2, p21^Waf1/Cip1^, p27^Kip1^, (**C**) pRb and E2F1 was determined by Western blot analysis using specific antibodies. The relative density of the protein was normalized to that of β-actin, which was used as an internal control. The mRNA expression of (**B**) cyclin D1, Cdk4, cyclin E, Cdk2, p27^Kip1^, (**D**) Rb, and E2F1 was quantified using qRT-PCR in BPH-1 cells. The relative mRNA expression was normalized using the Ct value of β-actin. The values represent as mean ± S.D. of three independent experiments. ** *p* < 0.01, *** *p* < 0.001 vs. vehicle control.

Progression of the cell cycle, comprising G1, S, G2, and M phases, is known to play a pivotal role in cellular responses, including cell proliferation [24]. Controlling the first checkpoint, the G1/S phase, is an essential step in cell cycle regulation, as cells are generally committed to the division following DNA replication [25]. Thus, we investigated the effect of UMB on the expression of the cyclin–Cdk complex that regulates the G1/S phase. In BPH-1 cells, overexpression of cyclin D1–Cdk4 and cyclin E–Cdk2 proteins was decreased following UMB treatment. In contrast, the protein expression of p21^Waf1/Cip1^ and p27^Kip1^, Cdk inhibitors, was increased by UMB treatment (Figure 2A). The qRT-PCR results showed that UMB treatment also suppressed the mRNA expression of cyclin D1–Cdk4 and cyclin E–Cdk2 while increasing the mRNA expression of p27^Kip1^ (Figure 2B). The transcription factor E2F1 and tumor suppressor protein Rb, the two major regulators of cell cycle progression, play a role in determining progression through the G1/S phase [26]. High expression of Rb phosphorylation (pRb) and E2F1 protein in BPH-1 cells, characterized by abnormal proliferation, was reversed by UMB treatment (Figure 2C). Likewise, the mRNA expression of Rb was upregulated, and that of E2F1 was downregulated by UMB treatment (Figure 2D).

### 2.3. Effect of UMB Treatment on E2F1 Expression in BPH-1 Cells

**Figure 3:**
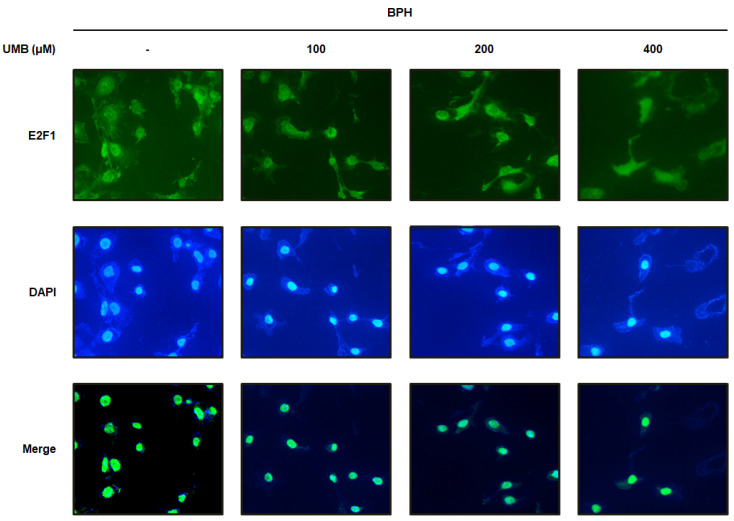
Effect of UMB treatment on E2F1 expression in BPH-1 cells. BPH-1 cells were treated with or without 100, 200, and 400 μM of UMB. Alexa Fluor 488-incorporated cells (green) were detected by immunofluorescence staining for E2F1. The nuclei were counterstained with DAPI (blue). Merged images represent staining for E2F1 and DAPI. The stained cells were visualized with a fluorescence microscope at 400× magnification.

Overexpression of E2F1 triggers uncontrolled cell proliferation leading to various diseases, including tumors [26]. Immunofluorescence analysis was performed to confirm the effect of UMB on E2F1 expression in BPH-1 cells. BPH-1 cells were identified by nuclear staining with DAPI, and E2F1-positive cells were detected as green fluorescence by Alexa Fluor 488. The large number of E2F1-positive cells visualized in BPH-1 cells was significantly suppressed by UMB treatment (Figure 3). These data support the hypothesis that the inhibitory effect of UMB on E2F1 expression is associated with the regulation of cell proliferation.

### 2.4. Effect of UMB Treatment on TGFβ1 and p-STAT3 (Tyr 705) Expression in BPH-1 Cells

**Figure 4:**
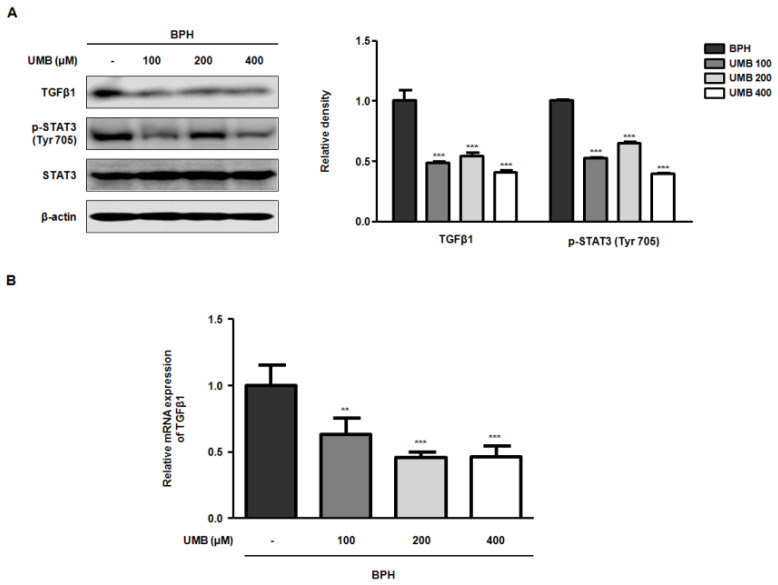
Effect of UMB treatment on TGFβ1 and p-STAT3 (Tyr 705) expression in BPH-1 cells. (**A**,**B**) BPH-1 cells were treated with or without 100, 200, and 400 μM of UMB. (**A**) Protein expression of TGFβ1 and p-STAT3 (Tyr 705) was determined by Western blot analysis using specific antibodies. The relative protein density was normalized to that of β-actin, which was used as an internal control. (**B**) The mRNA expression of TGFβ1 was quantified using qRT-PCR in BPH-1 cells. The relative mRNA expression was normalized using the Ct value of β-actin. The values represent as mean ± S.D. of three independent experiments. ** *p* < 0.01, *** *p* < 0.001 vs. vehicle control.

Transforming growth factor beta 1 (TGFβ1) encodes a protein that regulates many biological processes, including cell proliferation, differentiation, and growth [27]. STAT3, a member of the family of latent transcription factors, is activated by cytokines and growth factors to transcriptionally regulate the expression of genes involved in proliferation and survival [28]. TGFβ1 also enhances the nuclear localization of p-STAT3 by activating the STAT3 signaling pathway. Therefore, Western blot analysis and qRT-PCR were performed to determine whether UMB treatment of BPH-1 cells inhibited cell proliferation by blocking TGFβ1 and STAT3 activation. The protein expression of TGFβ1 and p-STAT3 (Tyr 705), which showed high expression in BPH-1 cells, was suppressed following UMB treatment (Figure 4A). Similarly, the mRNA expression of TGFβ1 was also significantly reduced following UMB treatment (Figure 4B). Therefore, these data suggest that UMB inhibits the proliferation of BPH-1 cells by impeding TGFβ1 and STAT3 activation.

### 2.5. Effect of UMB on Prostate Enlargement in TP-Induced Rats

**Figure 5:**
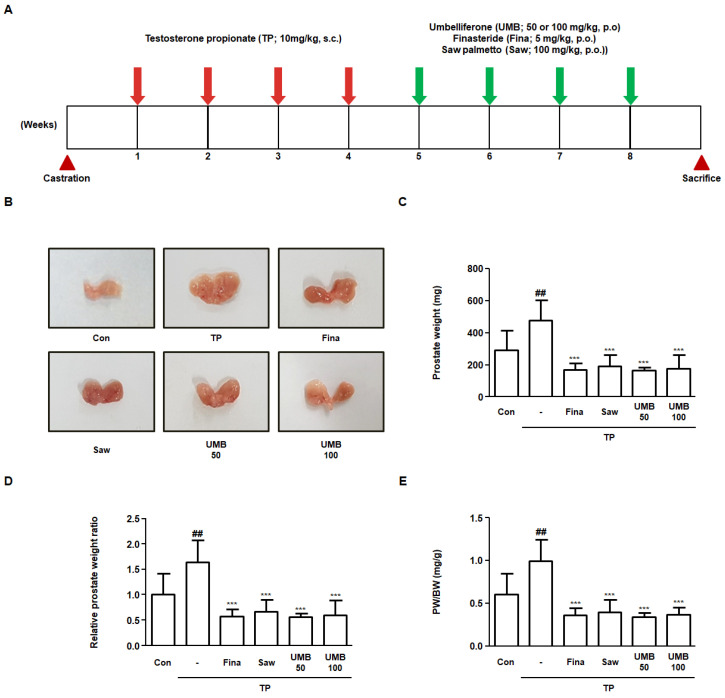
Effect of UMB on prostate enlargement in TP-induced rats. (**A**) Schematic overview of the experimental design of the in vivo study. (**B**) Visual comparison of the prostates of six experimental groups. (**C**–**E**) Comparative analysis of prostate weight in each group. (**C**) Prostate weight, (**D**) relative prostate weight ratio, and (**E**) prostate weight to body weight (PW/BW) ratio were measured. Relative prostate weight ratio was calculated by dividing the individual prostate weights of the experimental group by the mean prostate weight of the Con. The PW/BW ratio was calculated by dividing the prostate weight (mg) by the mean body weight (g). The data represent as mean ± S.D. of five rats per group. ^##^ *p* < 0.01 vs. Con group; *** *p* < 0.001 vs. TP group.

Rats with BPH were established by pre-injecting TP for four weeks, following which Fina (5 mg/kg), Saw (100 mg/kg), and UMB (50 or 100 mg/kg) were administered orally for four weeks (Figure 5A). To investigate the effect of UMB administration in vivo, the prostates were resected from the rats and analyzed. Visual comparison of prostate specimens from each group revealed that the prostate that had enlarged following TP following TP injection was reduced by administration of Fina, Saw, and UMB (50 or 100 mg/kg) (Figure 5B). Prostate weight was markedly increased in the TP group compared to Con group, whereas it was significantly decreased by administration of Fina, Saw, and UMB (50 or 100 mg/kg) (Figure 5C). Similarly, the relative prostate weight ratio was also elevated by TP, which was reduced following the administration of Fina, Saw, and UMB (50 or 100 mg/kg) (Figure 5D). The prostate weight to body weight ratio (PW/BW) was measured to determine whether the effect of UMB on prostate weight was affected by body weight. PW/BW showed a similar tendency to prostate weight, suggesting that prostate weight loss was not affected by body weight (Figure 5E).

### 2.6. Effect of UMB on Histological Alterations in TP-Induced Rats

**Figure 6:**
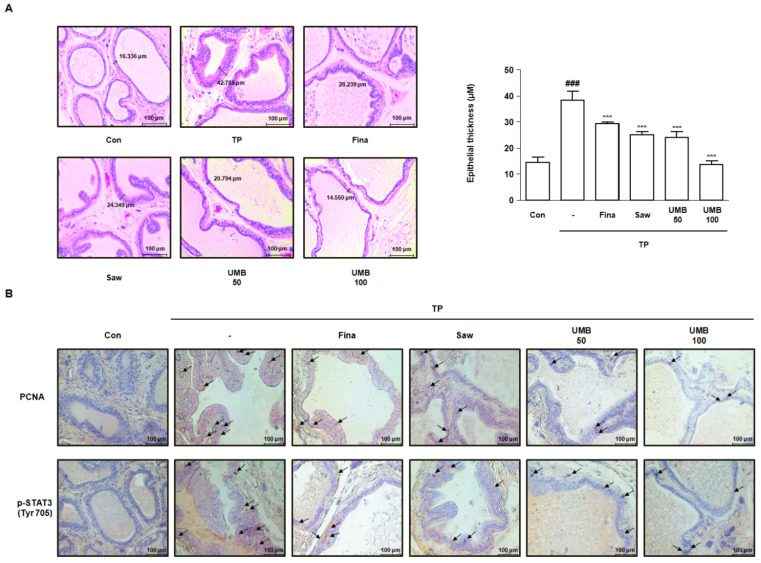
Effect of UMB on histological alterations in TP-induced rats. (**A**) Prostate tissue sections stained with hematoxylin and eosin (H&E) were observed using a Leica microscope (original magnification 20×). Epithelial thickness in the prostate tissues was measured and represented as the mean ± S.D. of five rats per experimental group. The expression of (**B**) PCNA and p-STAT3 (Tyr 705) was detected in prostate tissue of rats by immunohistochemical analysis using a Leica microscope (original magnification 20×). The black arrows indicate the immunoreactivity of PCNA and p-STAT3 (Tyr 705) highlighted in the prostate tissues of each group. ^###^ *p* < 0.001 vs. Con group; *** *p* < 0.001 vs. TP group.

To analyze the histological alterations in the prostate of TP-induced rats, hematoxylin and eosin (H&E) staining was performed to measure the epithelial thickness. The epithelial thickness, which was increased approximately 2.62-fold following TP injection, was significantly decreased following administration of Fina, Saw, and UMB (50 or 100 mg/kg) (Figure 6A). Furthermore, immunohistochemical analysis was performed to examine the expression of PCNA and STAT3 in relation to cell proliferation in prostate tissue. The expression of PCNA and p-STAT3 (Tyr 705) was highly detected in prostate tissue by TP injection, which was diminished by administration of Fina, Saw, and UMB (50 or 100 mg/kg) (Figure 6B). Therefore, we confirmed that the inhibitory effect of UMB on cell proliferation was related to the inhibition of STAT3 phosphorylation in TP-induced rats.

## 3. Discussion

The etiology of BPH has not yet been fully elucidated, but unregulated cell proliferation represents one of the major events in BPH progression. In addition, cell proliferation is closely associated with hormone disturbances, aging, and dysregulation of several growth factors [29]. Among these factors, increased androgen levels play a key role in the onset of BPH; therefore, treatment with 5ARI, including Fina, has been mainly used for patients with BPH [30]. However, long-term application of synthetic drugs such as Fina causes adverse effects, including impotence, loss of libido, hot flushes, fatigue, and psychological anxiety [31]. Hence, many studies are being conducted to identify synthetic drug alternatives from natural products with potential therapeutic effects and fewer side effects.

UMB is a naturally occurring comestible coumarin derivative of benzopyrone with a variety of biological activities. Its molecular structure is shown in Figure 1A. The presence of UMB in many medicinal plants and vegetables, along with its various pharmacological activities, emphasizes the utilization of UMB in the current study. Recently, Kandil et al. reported that 7-substituted UMB derivatives act as AR antagonists in the treatment of prostate cancer and breast cancer [32]. According to their results, the 7-substituted UMB derivative also inhibited cell proliferation in the human prostate cancer cell line, 22Rv1. The anti-cancer effect of UMB was further validated by Shen et al., who demonstrated that UMB treatment induces programmed cell death and cell cycle arrest in both early and late prostate cancer cells [22]. To the best of our knowledge, no previous studies have evaluated the effect of UMB on BPH. Thus, the present study sought to elucidate the anti-BPH effect of UMB and its underlying mechanisms in vitro and in vivo. Given that hormonal imbalance and pathological proliferation are the most prominent features of BPH, we constructed and used a human prostate epithelial cell line, BPH-1 (immortalized with SV-40 large T-antigen), and TP-induced rats subjected to orchiectomy for the study.

To understand how UMB inhibits aberrant proliferation of BPH-1 cells, the expression of genes related to the androgen/AR signaling pathway and PCNA were analyzed following UMB treatment. PCNA, a DNA polymerase delta auxiliary protein, is specifically expressed in the cell nucleus and is a significant marker for cell proliferation, synthesized in the G1/S phase of the cell cycle [33]. The overexpression of AR, SRC-1, PSA, and PCNA in BPH-1 cells was significantly inhibited by UMB treatment, indicating that UMB inhibits androgen/AR signaling-dependent cell proliferation (Figure 1). Altintas et al. reported that E2F1 directly interacts with AR in prostate cells and regulates cell proliferation by mediating the transcription of androgen-responsive gene expression [34]. The cyclin–Cdk complex promotes the pRb-mediated release of E2F1 in the late G1 phase, leading to the transcription of genes required for G1/S phase transition and DNA replication [35]. In contrast, p21^Waf1/Cip1^ and p27^Kip1^, members of the Cip/Kip family that are expressed in the G1 phase, play an important role in controlling pRb by inhibiting a wide range of Cdks, including Cdk4/6 and Cdk2 [36]. A study by Jiménez-Orozco et al. revealed that UMB exhibits a cytostatic effect by selectively reducing the percentage of cells expressing cyclin D1, which is consistent with the inhibition of the G1/S transition in the cell cycle [37]. Strikingly, our results also indicated that UMB treatment reduced the expression of cyclin D1–Cdk4 and cyclin E–Cdk2, while increasing the expression of p21^Waf1/Cip1^ and p27^Kip1^ (Figure 2A). Furthermore, UMB treatment prevented the dissociation of E2F1 by interfering with pRb (Figure 2B and Figure 3). Accordingly, the present study suggests that UMB inhibits abnormal cell proliferation through G1/S phase arrest of BPH-1 cells.

STAT3, which plays a critical role in the G1/S phase transition, was originally identified as a transcription factor activated by interleukin-6, and has subsequently been reported to transduce signals from additional cytokines, hormones, and growth factors [15]. Upstream kinases that regulate phosphorylation and transcriptional activation of STAT3, such as JAK2, JNK, and SRC, have been previously characterized as mediators of the non-canonical TGFβ signaling [38]. In this regard, the present study also demonstrated the inhibitory effect of UMB on TGFβ1 and p-STAT3 (Tyr 705) expression (Figure 4). Furthermore, to evaluate the effect of UMB in vivo, we established a rat model in which BPH was induced by pre-injecting TP subcutaneously for four weeks, followed by oral administration of UMB for the next four weeks. Administration of UMB (50 or 100 mg/kg) decreased the prostate weight, which was significantly increased by TP, similar to the positive controls Fina and Saw (Figure 5). These results are consistent with our histological analysis of the prostate. Epithelial thickness, which was markedly increased in the prostate of rats with BPH, was reduced following administration of Fina, Saw, and UMB (50 or 100 mg/kg) (Figure 6A). As an increased expression of PCNA and p-STAT3 (Tyr 705) correlates with cell proliferation, we further confirmed the inhibitory effect of UMB on cell proliferation by analyzing the expression of these markers in the prostate. The increased expression of PCNA and p-STAT3 (Tyr 705) in the prostate of rats with BPH was significantly decreased by administration of Fina, Saw, and UMB (50 or 100 mg/kg) (Figure 6B). Taken together, the present study demonstrated that the anti-BPH effect of UMB was mediated through inhibition of cell proliferation and G1/S phase cell cycle progression, both in vitro and in vivo.

## 4. Materials and Methods

### 4.1. Chemicals and Reagents

Fetal bovine serum (FBS) was obtained from Life Technologies Inc. (Grand Island, NY, USA). Penicillin-streptomycin solution was obtained from GE Healthcare Life Sciences Inc. (Chicago, IL, USA). Primary antibodies against PCNA (Cat. no. sc-56), AR (Cat. no. sc-816), SRC-1 (Cat. no. sc-32789), cyclin D1 (Cat. no. sc-753), Cdk4 (Cat. no. sc-23896), cyclin E (Cat. no. sc-481), Cdk2 (Cat. no. sc-748), p21^Waf1/Cip1^ (Cat. no. sc-397), p27^Kip1^ (Cat. no. sc-528), pRb (Cat. no. sc-377528), E2F1 (Cat. no. sc-193), TGFβ1 (Cat. no. sc-146), and β-actin (Cat. no. sc-47778) were obtained from Santa Cruz Biotechnology, Inc. (Dallas, TX, USA). Primary antibody against PSA (Cat. no. PB9259) was obtained from Boster Biological Technology (Pleasanton, CA, USA). Primary antibody against p-STAT3 (Tyr 705) (Cat. no. 9145) was obtained from Cell Signaling Technology (Danvers, MA, USA). Horseradish peroxidase-conjugated secondary antibodies were obtained from Jackson ImmunoResearch Laboratories, Inc. (West Grove, PA, USA). Oligonucleotide primers for PCNA, AR, SRC-1, PSA, cyclin D1, Cdk4, cyclin E, Cdk2, p27^Kip1^, Rb, E2F1, TGFβ1, and β-actin were purchased from Bioneer (Daejeon, Republic of Korea). Power SYBR^®^ green PCR master mix was obtained from Applied Biosystems (Foster City, CA, USA). Alexa Fluor 488 goat anti-rabbit IgG (H+L) was obtained from Invitrogen (Carlsbad, CA, USA). Testosterone propionate (TP) was procured from Wako Pure Chemicals Industries, Ltd. (Tokyo, Japan). Fina was obtained from Merck & Co., Inc. (Whitehouse Station, NJ, USA). Saw was purchased from Chong Kun Dang Healthcare Corp. (Seoul, Republic of Korea). All other reagents were acquired from Sigma-Aldrich (St. Louis, MO, USA).

### 4.2. Preparation of UMB and Cell Culture

UMB (Cat. no. H24003, purity 99%) was purchased from Sigma Chemical Co. (St. Louis, MO, USA) and dissolved in DMSO for the experiments. The stock solutions were stored at −20 °C. Human BPH-1 cells, representing epithelial cell types, were obtained from the Leibniz Institute DSMZ (Braunschweig, Lower Saxony, Germany). BPH-1 cells were maintained in RPMI 1640 medium (Gibco, Waltham, MA, USA) containing 20% FBS (Life Technologies Inc.) and 100 mg/mL penicillin-streptomycin (GE Healthcare Life Sciences Inc., Chicago, IL, USA) at 37 °C in a 5% CO_2_ incubator.

### 4.3. Cell Viability Assays

BPH-1 cells were plated in a 96-well plate at a concentration of 1 × 10^5^ cells/well and cultured overnight. The cells were treated with 6.25–400 μM UMB for 24 h. Then, the cells were incubated with MTT solution (5 mg/mL) for 4 h at 37 °C. The supernatant was aspirated, and the insoluble formazan crystals were dissolved in DMSO. The absorbance was measured at 540 nm using a BioTek Epoch microplate spectrophotometer (Winooski, VT, USA).

### 4.4. Western Blot Analysis

BPH-1 cells were homogenized in PRO-PREP™ protein extraction solution (Cat. no. 17081, iNtRON Biotechnology, Gyeonggi-do, Korea), and cell debris was removed by micro-centrifugation at 15,920× *g*, followed by quick freezing of the supernatants. Protein concentration was determined using the Bio-Rad protein assay reagent according to the manufacturer’s instructions (Bio-Rad, Hercules, CA, USA). Proteins were separated on a sodium dodecyl sulfate polyacrylamide gel and electroblotted onto a polyvinylidene fluoride membrane. The immunoreactive bands were detected by enhanced chemiluminescence (GE Healthcare Life Sciences Inc., Chicago, IL, USA), as described previously [39].

### 4.5. Isolation of Total RNA and Reverse Transcription Quantitative Polymerase Chain Reaction (RT-qPCR)

Total RNA was isolated from BPH-1 cells using an Easy-Blue RNA extraction kit (iNtRON Biotechnology, Inc., Gyeonggi, Korea), according to the manufacturer’s instructions. Total RNA samples were quantified using an Epoch^®^ microvolume spectrophotometer system (BioTek Instruments, Inc., Winooski, VT, USA). cDNA was obtained by reverse transcription using total RNA (1 μg), d(T)16 primer, and avian myeloblastosis virus reverse transcriptase (AMV-RT). Relative gene expression was measured using a Real-Time PCR System 7500 (Applied Biosystems, Foster City, CA, USA) with Power SYBR^®^ green PCR master mix. The primer sequences are listed in Table 1.

### 4.6. Immunofluorescence Staining

BPH-1 cells (1 × 10^5^ cells/mL) were cultured in a chamber slide (Lab-Tek II chamber slide #154526) for 24 h to detect E2F1. After treatment with UMB, the cells were fixed with 100% methanol for 30 min at room temperature and blocked with 10% normal goat serum (Gibco, Grand Island, NY, USA). The cells were incubated overnight with specific primary antibodies in a 10% blocking solution at 4 °C. After washing the primary antibodies with 0.3% Triton X in PBS, the cells were incubated with Alexa Fluor 488 goat anti-rabbit IgG (H+L) for 1 h. The nuclei were counterstained with 4′, 6-diamidino-2-phenylindole (DAPI, Life Technologies, Carlsbad, CA, USA) and observed under an optical microscope (ECLIPSE Ni-U, Nikon, Tokyo, Japan).

### 4.7. Animals

Eight-week-old male Wistar rats (200 ± 20 g) were obtained from Daehan Biolink Co. Ltd. (Daejeon, Korea) and maintained under steady conditions (light cycle: 12 h; temperature: 20–25 °C; humidity: 40–60%) with access to standard laboratory diet and tap water *ad libitum*. All procedures were conducted in accordance with the guidelines of the Institutional Animal Care and Use Committee of the Sangji University (approval number #2019-16). The rats were castrated to rule out the effect of intrinsic testosterone in all rats, except the sham-operated control group (Con) [40]. After a recovery period, BPH was induced by subcutaneous injections (s.c.) of TP (10 mg/kg/day) to the castrated rats, excluding Con, for four weeks. Rats with BPH were assigned to five groups (*n* = 5) and orally administered water (BPH), Fina (5 mg/kg, Fina), Saw (100 mg/kg, Saw), and UMB (50 or 100 mg/kg) for four weeks excluding the weekends.

### 4.8. Hematoxylin and Eosin (H&E) Staining and Immunohistochemistry (IHC)

Prostate tissues from the rats were fixed in 10% buffered formalin and embedded in paraffin. The tissue blocks were cut into 8 μm thickness and stained with H&E prior to histological evaluation. Images of the epithelial thickness were acquired using the Leica Application Suite software (LAS version 3.3.0, Leica Microsystems, Inc., Wetzlar, Germany). The tissue sections were placed onto microscopy slides (Muto Pure Chemicals Co., LTD., Tokyo, Japan), deparaffinized, and the endogenous peroxidase activity was depleted. The sections were then blocked with normal goat serum and incubated overnight at 4 °C with a primary antibody (1:200). The slides were then incubated with corresponding horseradish peroxidase-conjugated secondary antibody (1:500) and incubated at room temperature for 2 h. After washing thrice in PBS, the sections were incubated with 3,3′-diaminobenzidine (DAB) solution (Abcam, Cambridge, MA, USA). Finally, the tissues were counterstained with hematoxylin and mounted with mounting medium (Agilent Technologies, Inc., Santa Clara, CA, USA). Images were acquired using NIS-Elements F (version 4.0., Nikon, Tokyo, Japan).

### 4.9. Statistical Analysis

Data are expressed as the mean ± standard deviation (S.D.) of triplicate experiments. Significance was determined using Dunnett’s post hoc test for ANOVA using GraphPad Prism software (version 5.01, San Diego, CA, USA). Statistical significance was set at *p* < 0.05.

## 5. Conclusions

Our findings highlight the inhibitory effect of UMB on cell proliferation of BPH-1 cells and TP-induced rats. These effects appear to be mediated by blockade of the androgen/AR signaling pathway as well as G1/S phase cell cycle arrest through regulation of the E2F1/STAT3 axis. Moreover, UMB administration reduced the enlargement of the prostate in TP-induced rats, which was slightly superior to that observed with the conventional drugs, Fina and Saw. Thus, our findings suggest that UMB may be a potential therapeutic agent for the treatment of BPH.

## Figures and Tables

**Table 1:** Primer sequences.

Gene	Forward Primer (5′-3′)	Reverse Primer (5′-3′)
PCNA	TTAAACGGTTGCAGGCGTAG	AGGAAAGTCTAGCTGGTTTCGG
AR	GAGCCAGGTGTAGTGTGTGC	TCGTCCACGTGTAAGTTGCG
SRC-1	GCTGGTATCCTTCCTTAGTG	TGGCGTTGCTTGTTGTGGTG
PSA	ATAGGATTGCCCAGGCAGAA	CTAAGGGTAAAAGCAGGGAGAGAGT
Cyclin D1	ACGGCCGAGAAGCTGTGCATC	CCTCCGCCTCTGGCATTTTGGAG
Cdk4	ATGGCTACCTCTCGATATGAGC	CATTGGGGACTCTCACACTCT
Cyclin E	GACGGGGAGCTCAAAACTGA	TACAACGGAGCCCAGAACAC
Cdk2	TTCTATGCCTGATTACAAGCC	CTGGCTTGGTCACATCCT
p27^Kip1^	AACGTGCGAGTGTCTAACGG	CCCTCTAGGGGTTTGTGATTCT
Rb	ATGGTTCACCTCGAACACCC	TTTCGACACAACCCTGTCCC
E2F1	AAGAACCGCTGTTGTCCCG	TCGAGGCCGAAGTGGTAGTC
TGFβ1	CTATCGACATGGAGCTGGTGAAG	CGTGGAGCTGAAGCAATAGTTGG
β-actin	GGCCAGGTCATCACCATTGG	CTTTGCGGATGTCCACGTCA

## Data Availability

Not applicable.

## Abbreviations

AR	Androgen receptor
BPH	Benign prostatic hyperplasia
DHT	Dihydrotestosterone
E2F1	E2F transcription factor 1
Fina	Finasteride
PCNA	Proliferating cell nuclear antigen
PSA	Prostate-specific antigen
Saw	Saw palmetto
STAT3	Signal transducer and activator of transcription 3
TP	Testosterone propionate
TGFβ1	Transforming growth factor beta 1
UMB	Umbelliferone
